# A Case in Which Labor Was Retarded by a Large Tumor on the Nates of the Fetus

**Published:** 1853-10

**Authors:** John Bartlett

**Affiliations:** One of the Physicians to the Louisville Marine Hospital


Art. III.—*A Case in which Labor was Retarded by a Large Tumor on the Nates of the Fetus.* By John Bartlett, M.D., one of the Physicians to the Louisville Marine Hospital.

A. B., six months advanced in pregnancy, had been in labor since three o'clock, A.M. I saw her at nine. Os uteri as large as the palm of the hand; head presenting; every thing favorable. The labor advanced slowly. I ruptured the membranes when the os was fully dilated. The perineum was very gradually put on the stretch, and it was twelve o'clock before the head was born. The patient had six violent pains more, without the slightest advance of the child. I brought down the arms and made gentle traction to but little purpose. Afterwards, finding the cord tense, and thinking this might be the cause of delay, I delivered the legs, in doing which I detected a tumor growing from the buttock. The next pain, with my aid, expelled it.

*Fetus and Tumor.*—The fetus sits upon the tumor, which is three times as large as the head, round, and measures about thirteen inches in circumference. Its surface is every where smooth, and in front and on the sides plain ; behind it is nodulated. It is of a dark red color. The vulva is fully formed, and one half inch below, on the anterior surface of the tumor, is the anus, two and one half lines in diameter. The fetus has the appearance of a child sitting on a ten-pin ball.

The tumor looked very much as if it might be another fetus and placenta wrapped in membranes. On making an incision into it, some dark thin blood escaped. The bulk of the tumor consisted of a substance resembling crassamentum. The softer portions seemed to be held together by bands of red fibres running in various directions. Imbedded in the tumor were three or four masses of white, brain-like substance, about the consistence and color of the corresponding matter in the encephaloid. These white portions were an inch or two in diameter, irregular in shape, and emitted, on being opened, a grayish, frothy fluid, like scrofulous pus. The contents of the tumor were readily separable from the outer covering ; they might have been turned out and the cyst retained entire.

After the evacuation of the fluids the tumor diminished one-third in size. No other malformation or appearance of disease was noticeable. The abdominal organs were normal.

*Placenta.*—The placenta was large and white, and so soft as almost to fall to pieces. When placed on its fetal surface its cotyledons fell apart. It had evidently been macerated several days. There was an extraordinary quantity of liquor amnii, clear and not offensive. A pint of semi-coagulated blood escaped upon the extraction of the fetus. No flooding or other difficulty followed the labor.

*History of the Patient.*—The patient, 26 years of age, has always been healthy. She has had one child, which is well formed. She has been much less comfortable in this pregnancy than in her first. Her labor came on on Thursday; on the Saturday previous she complained of nausea, with pain in the back and limbs, and slight fever. These symptoms continued up to the time of her confinement. In this, as in her former pregnancy, she has had chills several times. She can assign no cause for her miscarriage. About two months before, she slipped off of a wood-pile and, as she says, hurt herself "a little."

September 10, 1853.

